# Metabolic Signatures in Lean MASLD: Current Insights and Future Directions

**DOI:** 10.3390/metabo15090583

**Published:** 2025-08-31

**Authors:** Ambrin Farizah Babu

**Affiliations:** 1 School of Medicine, Institute of Public Health and Clinical Nutrition, University of Eastern Finland, 70211 Kuopio, Finland; ambrin.babu@utu.fi; 2 Food Sciences Unit, Department of Life Technologies, University of Turku, 20014 Turku, Finland

**Keywords:** lean metabolic dysfunction-associated steatotic liver disease, non-alcoholic fatty liver disease, body mass index, metabolomics, lipidomics, gut microbial metabolites, genetic and epigenetic modifiers

## Abstract

Lean metabolic dysfunction-associated steatotic liver disease (lean MASLD) challenges longstanding views that link hepatic steatosis primarily to obesity. Emerging as a distinct and under-recognized clinical entity, lean MASLD affects individuals with a normal body mass index (BMI), yet carries risks of cardiovascular disease, hepatocellular carcinoma, and liver-related mortality comparable to obesity-associated MASLD. The absence of overt metabolic dysfunction complicates diagnosis, revealing critical limitations in current screening frameworks centered on BMI. This review synthesizes evolving clinical insights and epidemiological trends in lean MASLD, and delineates its unique pathophysiological mechanisms. Recent advances in metabolomics have uncovered disease-specific disruptions in lipid and amino acid metabolism, bile acid signaling, and gut microbiota-derived metabolites. By integrating evidence from metabolic, genetic, and epigenetic domains, we identified promising biomarkers, and therapeutic targets that may support earlier detection and precision-guided treatment strategies.

## 1. Introduction

Metabolic dysfunction-associated steatotic liver disease (MASLD), previously known as non-alcoholic fatty liver disease (NAFLD), represents a broad spectrum of liver diseases characterized by the accumulation of fat in the liver in the absence of significant alcohol consumption [1]. This spectrum ranges from simple hepatic steatosis to more advanced stages such as steatohepatitis, fibrosis, cirrhosis, and hepatocellular carcinoma [1]. While obesity is a well-established risk factor for MASLD [2], growing evidence highlights a distinct subset of patients with lean MASLD who develop hepatic steatosis despite having a normal body mass index (BMI) [3]. This phenotype challenges the conventional view that adiposity is the primary driver of liver fat accumulation. Although lean individuals may lack overt obesity, they often exhibit subtle metabolic dysfunctions, ectopic fat deposition, and increased cardiometabolic risk [3].

The rise in lean MASLD underscores the need for more precise diagnostic tools and a better understanding of underlying mechanisms. Currently, approximately 10–15% of MASLD patients are classified as lean, and this phenotype has been associated with cardiovascular and cancer-related mortality risks comparable to those of obese MASLD patients, as well as elevated all-cause mortality [4,5].

Understanding the distinct pathophysiological features of lean MASLD is essential for developing targeted strategies for diagnosis, risk stratification, and treatment. This underscores the importance of moving beyond BMI as a sole metric for assessing liver disease risk and embracing a more nuanced perspective on metabolic health. Metabolomics, the comprehensive profiling of small-molecule metabolites in biological systems, has emerged as a powerful approach to capture the dynamic metabolic changes associated with lean MASLD [6].

By offering a systems-level snapshot of metabolic perturbations, metabolomics enables the identification of disease-specific biomarkers, disrupted pathways, and potential therapeutic targets. In the context of lean MASLD, where conventional risk markers may be absent or misleading, metabolomics holds promise in unraveling hidden biochemical signatures that drive disease onset and progression.

This review explores the current insights into the metabolic signatures of lean MASLD, with a focus on lipid metabolism, amino acids, bile acids (BAs), gut microbial metabolites, and genetic and epigenetic modifiers. It also highlights emerging metabolomic findings, their diagnostic and therapeutic implications, and future research directions necessary to better understand and manage this underrecognized but clinically significant phenotype.

## 2. Epidemiology, Clinical Characteristics, and Diagnostic Implications

The prevalence of lean MASLD varies significantly across geographic regions, reflecting differences in genetic, lifestyle, and environmental factors. According to recent global estimates, the prevalence of lean MASLD is approximately 5.1% [7], with lean MASLD being particularly common in Asian populations [7]. Cross-sectional studies estimate that 7–20% of individuals with MASLD have a lean body [8]. Notably, the prevalence of lean MASLD has shown a rising trend over the past few decades, increasing steadily between 1988 and 2017 [9]. In Asia, the prevalence among middle-aged adults (45–59 years) reached 4.4% [9], while a magnetic resonance spectroscopy-based study in Hong Kong reported hepatic steatosis in 19% of non-obese individuals compared to 61% in obese individuals [10]. Ethnic disparities are also evident in the expression of lean MASLD. For instance, lean, non-diabetic, non-smoking Asian Indians have demonstrated a twofold higher prevalence of hepatic steatosis compared to BMI-matched Caucasians, Hispanics, Blacks, and East Asians, highlighting a disproportionate metabolic burden in this group [11]. In Western populations, lean MASLD is also gaining attention, with prevalence estimates of up to 16% in Italy [12] and 9.3%% in the United States [13].

Clinically, lean MASLD is diagnosed in individuals with hepatic steatosis and a BMI <25 kg/m^2^ in Asians or <30 kg/m^2^ in non-Asians, alongside at least one cardiometabolic risk factor [14]. Those with coexisting significant alcohol intake are reclassified under metabolic and alcohol-related liver disease. Due to its subtle presentation, lean MASLD frequently goes undetected, and most cases are discovered incidentally through imaging or during routine evaluations. Liver enzymes are typically normal or only mildly elevated, and symptoms are vague or nonspecific [14]. Contrary to earlier assumptions that lean individuals are metabolically healthy, it is now evident that lean MASLD often presents with significant metabolic complications, including visceral adiposity, insulin resistance, dyslipidemia, and hypertension despite a normal BMI. Subgroup analyses have further revealed a higher incidence of metabolic syndrome among females in the lean MASLD population, suggesting sex-specific vulnerabilities [7].

Currently, there are no specific guidelines tailored to the diagnosis of lean MASLD, and much of the existing diagnostic framework has been extrapolated from studies on obese populations. A comprehensive diagnostic workup is critical for accurate assessment and appropriate management. While abdominal ultrasonography is a widely used, non-invasive tool for detecting hepatic steatosis, it provides limited insight into liver fibrosis, i.e., the key predictor of liver-related and cardiovascular outcomes [15]. Several non-invasive serum-based scoring systems, such as the Fibrosis-4 Index (FIB-4) and the NAFLD Fibrosis Score (NFS), are commonly used in MASLD populations to estimate fibrosis risk [16]. However, these scores have not been specifically validated in lean individuals, and their accuracy in this subgroup remains uncertain [16]. In cases with indeterminate risk or discordant findings, imaging modalities such as transient elastography (FibroScan) or magnetic resonance elastography (MRE) are recommended for further fibrosis assessment [17,18,19]. Liver biopsy remains the gold standard but is reserved for diagnostic uncertainty or when non-invasive tests yield inconclusive results [15].

Metabolomics has emerged as a transformative tool in the diagnosis and management of lean MASLD. By identifying disease-specific metabolic fingerprints in blood, urine, or feces, metabolomics allows for the earlier detection of molecular alterations preceding structural liver damage. Distinct patterns involving lipid metabolism, amino acid catabolism, bile acid composition, etc., have been identified in lean MASLD, offering potential for non-invasive diagnosis, disease stratification, and therapeutic monitoring [6].

## 3. Mechanistic Insights into Lean MASLD

The mechanisms driving the development of lean MASLD are less well understood than those of non-lean MASLD, reflecting a complex interplay of metabolic, genetic, and microbiome-related factors. Lean MASLD potentially arises from a convergence of subtle hepatic stressors, including disturbances in cellular energy sensing, impaired mitochondrial resilience, and dysregulated immune signaling [8,20]. While chronic oxidative stress, impaired autophagy, and disrupted hepatocyte signaling networks have been well documented as drivers of inflammation and early fibrotic remodeling in classical MASLD [21,22], similar mechanistic studies in the lean phenotype remain limited.

Recent studies indicate that lean MASLD is associated with disproportionate visceral fat accumulation and ectopic lipid storage in the liver, despite a normal BMI [23]. This atypical fat distribution, coupled with sarcopenia, i.e., the loss of skeletal muscle mass and function, is further known to exacerbate insulin resistance and systemic metabolic dysfunction [24]. Genetic predispositions add another layer of complexity, as variants affecting lipid metabolism, inflammation, and susceptibility to oxidative stress modulate individual risk [25]. These genetic factors may help explain why fibrosis and liver injury can be more pronounced in lean MASLD patients compared with their non-lean counterparts. Gut microbiota alterations constitute an additional mechanistic layer in lean MASLD pathogenesis. Changes in microbial composition can disrupt bile acid metabolism and increase intestinal permeability, promoting hepatic inflammation and systemic metabolic derangements [26]. These gut–liver interactions are particularly relevant in individuals with coexisting inflammatory bowel disease, where chronic intestinal inflammation and elevated pro-inflammatory cytokines amplify hepatic steatosis and contribute to disease progression [3]. Beyond hepatic consequences, lean MASLD exerts systemic effects that elevate cardiovascular risk. Even in the absence of obesity, patients exhibit endothelial dysfunction, dyslipidemia, and systemic inflammation, all of which accelerate atherosclerosis and increase morbidity [27]. This paradoxical phenotype highlights that conventional markers of metabolic syndrome may underestimate risk in lean individuals, emphasizing the need for targeted assessment of mechanistic pathways and early biomarker identification.

## 4. Metabolic Signatures in Lean MASLD

### 4.1. Lipid Metabolism

Individuals with lean MASLD exhibit distinct alterations in lipid metabolism compared to their obese counterparts. Serum or plasma profiling consistently shows that levels of triglycerides (TG), low-density lipoprotein cholesterol (LDL-C), alanine aminotransferase (ALT), and gamma-glutamyl transferase (GGT) are generally lower in lean MASLD patients than in those with obesity-associated MASLD, yet remain elevated when compared to lean or obese individuals without liver disease [7]. However, these levels remain higher than those observed in both lean and obese non-MASLD individuals [7]. Interestingly, lean MASLD patients tend to exhibit the highest levels of total cholesterol (TC) and alkaline phosphatase (ALP), pointing to unique lipid regulatory disturbances in this subgroup [7]. ALT, a cytoplasmic enzyme released during hepatocyte injury, reflects the integrity of liver cells and is elevated in lean MASLD, likely due to lipid peroxidation-driven oxidative stress [28]. This elevation, along with increased TG levels compared to lean healthy controls, underscores the role of excess hepatic lipid accumulation in driving hepatocellular damage and inflammation. The dysregulation of lipid transport proteins further contributes to disease progression; for example, APOA-1, a key apolipoprotein involved in high-density lipoprotein (HDL)-mediated cholesterol transport, is reduced in lean MASLD, impairing reverse cholesterol transport and promoting hepatic lipid retention [28].

**Figure 1:**
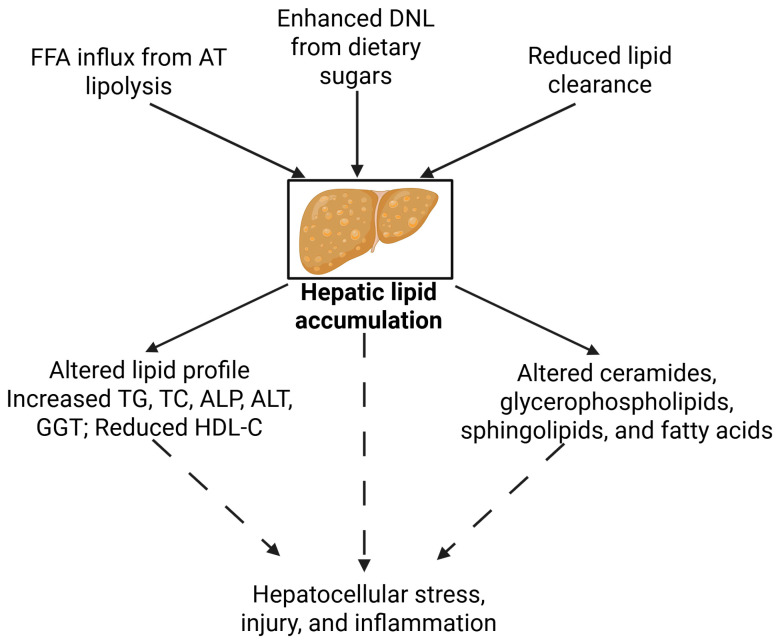
Altered lipid metabolism in lean MASLD.

At the mechanistic level, hepatic lipid accumulation in lean MASLD is driven by a combination of increased free fatty acid (FFA) influx from adipose tissue lipolysis, enhanced de novo lipogenesis (DNL) from dietary sugars, and reduced lipid clearance via mitochondrial β-oxidation or very low-density lipoprotein (VLDL) secretion [29] (Figure 1, Table 1). These alterations are often associated with an atherogenic lipid profile, characterized by elevated levels of small, dense low-density lipoprotein particles and reduced concentrations of HDL [29]. This pro-atherogenic profile contributes to an increased risk of cardiovascular disease, a major comorbidity in MASLD [30,31]. In lean individuals, the development of hepatic steatosis, despite a normal BMI, is largely attributed to increased DNL, which is upregulated in response to insulin resistance and hyperinsulinemia [32]. This process leads to the accumulation of lipotoxic lipid species and contributes to hepatocellular stress [32]. As a result, both the quantity and composition of hepatic lipid species are altered, with elevated levels of palmitic acid (PA), oleic acid (OA), linoleic acid (LA), and arachidonic acid (AA) frequently observed [28]. These fatty acids contribute to cellular lipotoxicity, mitochondrial dysfunction, and endoplasmic reticulum stress [28]. Notably, PA and OA are associated with hepatocyte steatosis and inflammation [33], while elevated LA and AA, especially as the downstream products of essential fatty acid metabolism, are linked to both early stages and disease progression through inflammatory pathways [34,35]. In addition, several glycerophospholipids, including lysophosphatidylcholines LPC 18:0, LPC 17:0, and PC 36:3, were altered in lean MASLD subjects compared to lean healthy and obese MASLD counterparts [23]. Moreover, the accumulation of ceramides, a class of pro-inflammatory sphingolipids, has been documented in lean MASLD, and is implicated in the development of insulin resistance and hepatocellular injury [14]. Notably, several sphingomyelins, including 24:1, 14:1, 22:1, and 22:2, were also found to be altered in lean MASLD compared to healthy controls [23]. As the downstream products of ceramides, sphingomyelins share biosynthetic pathways and functional overlap, and have been associated with insulin resistance and cardiovascular risk [36]. Furthermore, alterations in acylcarnitines have also been reported in lean MASLD. While short-chain acylcarnitines such as C3 and C4 were lower in lean MASLD than in obese MASLD, they resembled those in lean controls, suggesting these changes may be obesity-specific [23]. In contrast, medium-chain acylcarnitines such as C5 acylcarnitine were elevated, and C8 acylcarnitine was reduced in lean MASLD, pointing toward disruptions in mitochondrial fatty acid and amino acid metabolism [23], suggesting that lean MASLD is not merely a milder version of obesity-driven disease, but a metabolically distinct phenotype with unique diagnostic and therapeutic implications.

### 4.2. Amino Acid Metabolism

**Figure 2:**
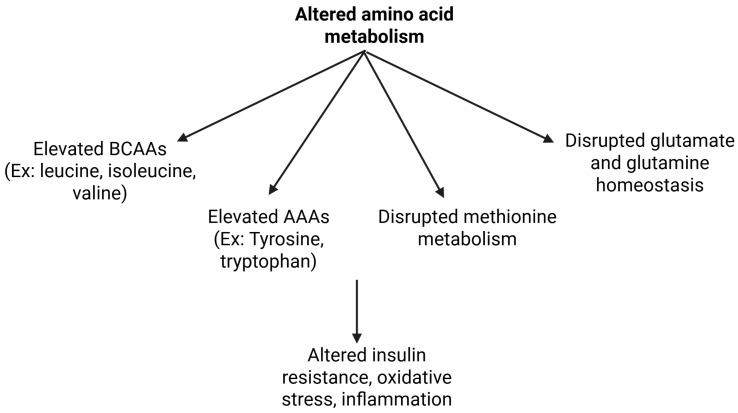
Altered amino acid metabolism in lean MASLD.

Emerging evidence suggests that disturbances in amino acid metabolism are a central feature of MASLD [6] (Figure 2, Table 1). One of the most consistently reported features is the elevation in branched-chain amino acids (BCAAs: leucine, isoleucine, and valine), which are strongly associated with insulin resistance, hepatic lipid accumulation, and systemic metabolic dysfunction [37]. While elevated BCAA levels have been extensively documented in obesity-associated MASLD, their persistence in lean individuals suggests an underlying metabolic inflexibility independent of adiposity. This points toward an intrinsic hepatic and systemic metabolic disturbance that may contribute to steatosis even in the absence of excess fat mass. Mechanistically, impaired BCAA catabolism in hepatic and peripheral tissues leads to the accumulation of toxic metabolic intermediates and aberrant activation of the mTOR signaling pathway, which promotes lipid dysregulation, inflammation, and insulin resistance [38]. Supporting this, Muyyarikkandy et al. identified a distinct metabolic signature in lean MASLD, where altered triglyceride and BCAA metabolism aggravated mitochondrial dysfunction via suppression of the AMPK pathway, ultimately contributing to hepatocellular injury and elevated ALT levels [39]. However, contrasting evidence from Feldman et al. [40] suggests that BCAA elevations may not be a universal feature of MASLD across phenotypes. Their study found that BCAAs were significantly elevated only in MASLD-obese individuals, while obese and lean healthy controls did not exhibit similar changes, indicating that BCAA accumulation may be more reflective of hepatic metabolic dysfunction rather than obesity or leanness alone [40].

In parallel, increased levels of aromatic amino acids (AAAs), particularly tyrosine, have been observed in lean MASLD, suggesting compromised hepatic clearance and altered amino acid flux [23]. Elevated AAAs have also been linked to insulin resistance and changes in neurotransmitter metabolism, which may indirectly impact appetite regulation and systemic energy homeostasis [41].

Disruptions in methionine metabolism and the one-carbon cycle have also gained attention. Altered levels of S-adenosylmethionine (SAM) and S-adenosylhomocysteine (SAH) indicate impaired methylation capacity and reduced glutathione synthesis, contributing to oxidative stress, hepatocellular injury, and fibrosis [42]. Elevated homocysteine further links this dysregulation to cardiovascular and liver disease risk [43]. Notably, plasma SAM—but not methionine—is independently associated with increased fat mass and truncal adiposity, reflecting enhanced methionine-to-SAM conversion in obesity [44]. Animal protein intake correlates positively with plasma SAM and cysteine levels, suggesting dietary influence on this pathway.

**Table 1:** Key metabolic and molecular features of lean MASLD.

Category	Alteration	Key Metabolite/Mechanism	Implications	References
**Lipid Metabolism**	↑ De novo lipogenesis ↓ APOA1↑ Triglycerides ↑ Total cholesterol, ALP	- Elevated fatty acids - Increased ceramides - Increased glycerophospholipids (e.g., LPC 18:0, PC 36:3)	Lipotoxicity, ER stress, mitochondrial dysfunction, hepatocellular damage	[23,28,32]
**Amino Acid Metabolism**	↑ BCAAs (Leu, Ile, Val) ↑ Aromatic AAs (e.g., tyrosine) ↑ SAM, SAH ↓ Glutamine/glutamate ratio	- Impaired BCAA oxidation, mTOR activation - One-carbon cycle dysfunction - Elevated GSG index - Kynurenine pathway activation (↑ Kyn/Trp)	Insulin resistance, oxidative stress, hepatic inflammation, early fibrosis	[37,41,42]
**Bile Acid and Gut Microbial Metabolites**	↑ Secondary BAs↑ FGF19 ↑ SCFAs (isobutyrate, propionate) ↓ 7α-hydroxy-4-cholesten-3-one	- Increased thermogenic BAs (e.g., cholic acid) - SCFA-specific changes in lean MASLD	Gut–liver axis dysregulation, altered energy metabolism, hepatic inflammation	[45,46]
**Genetic Modifiers**	↑ PNPLA3 (GG), TM6SF2 (TT)PEMT (Val175Met variant)	- Reduced lipid export, phospholipid imbalance - Epigenetic risk via DNA methylation	Genetic predisposition in lean phenotypes	[25]
**Epigenetics and miRNAs**	↓ Histone variants (macroH2A1.1, 1.2) ↑ miR-4488	- Gene silencing of protective regulators		[47,48]

Glutamine and glutamate homeostasis is frequently disrupted in MASLD, with several studies reporting a decreased glutamine/glutamate ratio [49]. This imbalance likely reflects enhanced hepatic glutaminolysis, impaired ammonia detoxification, and altered TCA cycle intermediates, contributing to mitochondrial dysfunction and inflammation [49]. The glutamate–serine–glycine (GSG) index is elevated in MASLD, with higher levels observed in obese compared to lean individuals [50]. Furthermore, an increased glutamate/glutamine ratio correlates with fibrosis severity, as demonstrated by Du et al. [51], who also showed that 18F-fluoroglutamine positron emission tomography (PET) imaging detects heightened hepatic tracer uptake in fibrotic mice, suggesting its potential as a non-invasive marker of early fibrogenic activity.

The tryptophan metabolic pathway, regulated by inflammation-responsive enzymes such as indoleamine 2,3-dioxygenase (IDO1), is upregulated in MASLD [52]. Elevated kynurenine-to-tryptophan ratios, indicative of increased IDO1 activity, reflect a pro-inflammatory state that may worsen liver injury and drive progression to steatohepatitis [52]. Notably, this ratio is higher in obese compared to lean individuals with MASLD [53]. Experimental indole supplementation in both lean and obese mice has been shown to reduce macrophage infiltration and pro-inflammatory markers, underscoring the therapeutic potential of targeting this pathway to alleviate hepatic inflammation [54].

In addition, elevated levels of urea nitrogen and uric acid have been reported in lean MASLD patients compared to healthy controls [28], indicating altered nitrogen disposal and purine metabolism. These changes may reflect increased amino acid turnover and hepatic stress, further linking nitrogen handling with metabolic dysfunction in lean MASLD.

### 4.3. Bile Acids and Gut Microbial Metabolites

Metabolites such as secondary bile acids and short-chain fatty acids (SCFAs) are perturbed in lean MASLD, reflecting gut–liver axis dysregulation (Table 1). Patients with lean MASLD exhibit higher serum levels of secondary BAs and fibroblast growth factor 19 (FGF19), along with reduced 7-alpha-hydroxy-4-cholesten-3-one, an intermediate in BA synthesis [45]. These changes are more pronounced in early fibrosis stages, suggesting a dynamic metabolic adaptation. This phenotype is in line with the findings in lean murine models, where BA transporter inhibition shifts BA profiles and gut microbiota, improving steatohepatitis [45].

Elevated BA levels in lean MASLD may contribute to an obesity-resistant phenotype by increasing energy expenditure [55]. Key BAs, including cholic acid, deoxycholic acid, and chenodeoxycholic acid, enhance thermogenesis and brown adipose tissue activity [56,57]. FGF19, also increased in lean MASLD, regulates energy balance and glucose-lipid homeostasis, with gut-restricted FXR agonists promoting metabolic improvements and adipose tissue browning in mice [58]. Despite increased BA levels, no direct association with hepatic steatosis was observed, implying peripheral metabolic effects or microbiota-mediated mechanisms may drive fat accumulation in the liver [45]. Insulin resistance in lean MASLD activates lipogenic transcription factors—SREBP-1c and ChREBP—enhancing de novo lipogenesis, compounded by BA dysmetabolism and cholestasis, as evidenced by elevated alkaline phosphatase and gamma-glutamyl transferase [59].

SCFAs such as isobutyrate and propionate are markedly increased in lean MASLD but not in obese MASLD, suggesting differential microbial fermentation profiles [46]. These metabolites may influence hepatic lipid metabolism and inflammation.

### 4.4. Other Metabolites

Metabolomics analysis of the stool samples have identified distinct metabolic alterations in non-obese MASLD, including elevated tyramine glucuronide, 9,12,13-trihydroxyoctadecenoic acid (TriHOME), and pantetheine 4′-phosphate, alongside reduced levels of bacterial metabolites such as 3-carbamoyl-2-phenylpropionaldehyde and N-succinyl-L,L-2,6-diaminopimelate, as well as homogentisic acid and estriol [60]. These changes reflect disruptions in lipid metabolism—particularly linoleic acid pathways—gut microbial alterations, and hormonal imbalances. Tyramine glucuronide and TriHOME are linked to hepatotoxicity and lipid dysregulation [61], while pantetheine 4′-phosphate may enhance fatty acid synthesis, promoting disease progression [62]. Decreased bacterial metabolites suggest altered gut microbiota and toxin metabolism, and fluctuating estrogen metabolites underscore estrogen’s complex role in liver lipid homeostasis [60].

## 5. Genetic and Epigenetic Modifiers

Genetic and epigenetic factors play crucial roles in the pathogenesis of lean MASLD, distinguishing it from obesity-associated forms (Table 1). Polymorphisms in genes such as PNPLA3 and TM6SF2 are more prevalent in lean MASLD, and influence lipid handling and inflammation [25]. For example, individuals with the PNPLA3 GG genotype exhibit a markedly higher risk of MASLD when lean compared to those who are overweight or obese, relative to the CC genotype. Similarly, the TM6SF2 rs58542926 TT genotype correlates with reduced serum triglyceride levels specifically in lean individuals [25]. In contrast, no significant gene–BMI interactions have been observed for other polymorphisms, including MBOAT7 rs626283 and several other single-nucleotide polymorphisms [25].

Epigenetic mechanisms play a significant role in the development and progression of lean MASLD by regulating gene expression without altering DNA sequences. These mechanisms include DNA methylation, histone modification, and microRNA, all of which are influenced by environmental and metabolic factors [63]. DNA methylation, particularly involving the PEMT gene, has been linked to increased susceptibility to lean MASLD. A specific variant, Val175Met, has been identified as a key risk factor in the Japanese population, where it impairs PEMT enzyme activity, disrupts phospholipid metabolism, and contributes to hepatic fat accumulation, ultimately increasing the risk of progression to MASH [64]. PEMT knockout mice exhibit fatty liver phenotypes without obesity, closely resembling human lean MASLD [63]. Further, histone modifications, especially the depletion of histone variants macroH2A1.1 and macroH2A1.2, have been observed in lean MASLD subjects [47]. Additionally, miRNAs are single-stranded, noncoding RNAs consisting of 20–24 nucleotides that play a crucial role in post-transcriptional gene regulation by modulating protein translation. Their dysregulation can significantly influence the onset and progression of various pathophysiological conditions, including metabolic liver diseases [65]. In the context of lean MASLD, miRNAs are particularly important due to their role in regulating lipid and carbohydrate metabolism [66]. Notably, serum miR-4488 has emerged as a promising noninvasive biomarker for the early detection of lean MASLD, with significantly elevated levels observed in lean MASLD patients compared to non-lean MASLD individuals and healthy controls [48].

## 6. Future Research Directions

Future research should prioritize effective screening strategies for MASLD, especially given its role as a precursor to the more severe steotohepatitis and the rising prevalence of lean MASLD. Current screening guidelines focus mainly on high-risk groups such as obese individuals and those with type 2 diabetes, leaving lean individuals underdiagnosed [67]. Longitudinal metabolomic studies are needed to monitor disease progression over time, while multi-omics approaches integrating genomics, transcriptomics, proteomics, and metabolomics can provide a comprehensive understanding of lean MASLD pathogenesis. Research must also include ethnically diverse cohorts to capture population-specific variations and identify therapeutic targets tailored to lean MASLD. Additionally, investigations focusing on pediatric and adolescent populations are essential to address early onset and prevention strategies in lean MASLD.

## 7. Conclusions

Lean MASLD is a metabolically complex and clinically significant condition. Metabolomics provides a window into the unique pathophysiology of this phenotype, offering opportunities for early diagnosis and targeted therapy. The metabolic perturbations not only provide insight into disease mechanisms, but also offer promising avenues for biomarker discovery and targeted interventions, particularly dietary strategies aimed at restoring metabolic homeostasis and enhancing hepatic resilience. Future research should aim to unravel the interplay between metabolites, gut microbiota, and genetic predispositions to fully characterize the lean MASLD phenotype. Future research should aim to unravel the metabolic underpinnings of lean MASLD and translate these findings into clinical practice.

## Data Availability

No new data were created or analyzed in this study.

## Abbreviations

The following abbreviations are used in this manuscript:

MASLD	Metabolic Dysfunction-Associated Steatotic Liver Disease
NAFLD	Non-alcoholic fatty liver disease
BMI	Body mass index
BA	Bile acid
FIB-4	Fibrosis-4 Index
NFS	NAFLD Fibrosis Score
MRE	Magnetic resonance elastography
TG	Triglycerides
LDL-C	Low-density lipoprotein cholesterol
ALT	Alanine aminotransferase
TC	Total cholesterol
ALP	Alkaline phosphatase
HDL	High-density lipoprotein
FFA	Free fatty acid
DNL	De-novo lipogenesis
VLDL	Very low-density lipoprotein
PA	Palmitic acid
OA	Oleic acid
LA	Linoleic acid
AA	Arachidonic acid
LPC	Lysophosphatidylcholine
BCAA	Branched-chain amino acid
AAA	Aromatic amino acid
SAM	S-adenosylmethionine
SAH	S-adenosylhomocysteine
GSG	Glutamate–serine–glycine
IDO1	Indoleamine 2,3-dioxygenase
PET	Positron emission tomography
SCFA	Short-chain fatty acid
FGF19	Fibroblast growth factor 19
TriHOME	9,12,13-trihydroxyoctadecenoic acid
